# Decoding hotline’s information with text-mining: A protocol for improving tobacco control in Shanghai

**DOI:** 10.18332/tid/187864

**Published:** 2024-06-17

**Authors:** Tong Zhao, Zi-an He, Jiaqi Shao, Aksara Regmi, Lili Shi, Yuyang Cai

**Affiliations:** 1 School of Public Health, Shanghai Jiao Tong University School of Medicine, Shanghai, China; 2 Jiading District Center for Disease Control and Prevention, Shanghai, China; 3 Zhongshan Hospital, Fudan University, Shanghai, China; 4 Xinhua Hospital, Shanghai Jiao Tong University School of Medicine, Shanghai, China

**Keywords:** hotline, text-mining, smoking ban, audience-centered policymaking

## Abstract

Tobacco consumption in China remains the primary cause of preventable mortality, with Shanghai being particularly affected by issues related to secondhand smoke exposure. This study explores the role of the public service hotline 12345, a grassroots initiative in Shanghai, in capturing public sentiment and assessing the effectiveness of anti-smoking regulations. Our research aims to accurately and deeply understand the implementation and feedback of smoking control policies: by identifying high-frequency points and prominent issues in smoking control work based on the smoking control work order data received by the health hotline 12320. The results of this study will assist government enforcement agencies in improving smoking monitoring and clarify the direction for improving smoking control measures. Text-mining techniques were employed to analyze a dataset comprising 78011 call sheets, all related to tobacco control and collected from the hotline between 1 January 2015 and 31 December 2019. This methodological approach aims to uncover prevalent themes and sentiments in the public discourse on smoking and its regulation, as reflected in the hotline interactions. Our study identified hotspots and the issues of greatest concern to citizens. Additionally, it provided recommendations to enforcement agencies to enhance their capabilities, optimize the allocation of human resources for smoking control monitoring, reduce enforcement costs and support for anti-smoking campaigns, thereby contributing to more effective tobacco control policies in the region.

## INTRODUCTION

China is the world’s largest producer and consumer of tobacco^1^, where a staggering 44% of the world’s cigarettes are smoked^2^. In 2009, China produced 2.3 trillion cigarettes, accounting for 40% of the world’s total^1^. Smoking is a major risk factor for mortality in China. Continued strengthening of national programs and initiatives for smoking prevention and cessation is needed to reduce smoking-related deaths in China^3^. Comprehensive smoke-free legislation is an effective way to prevent the hazards of passive smoking^4^. In order to protect the public from the harmful effects, Shanghai officially carried out the ‘Regulations of Shanghai Municipality on smoking control in public places’ (referred to as the Regulation) on 1 March 2010. On 11 November 2016, the Standing Committee of the Shanghai Municipal People’s Congress revised the Regulation and issued the ‘decision on amending the Regulations of Shanghai Municipality on smoking control in public places’ (referred to as the Amendment). Carried out on 1 March 2017, the Amendment clearly pointed out that ‘smoking is prohibited in indoor public places, indoor workplaces and public transportation’^5^. Compared with previous policies, the Amendment extended the ban on smoking from parts of indoor places to citywide indoor public places, indoor workplaces, and public transportation. At the same time, the public service hotline 12345 was introduced into the tobacco control monitoring plan, marking a giant step forward to creating smoke-free environments^4^.

The hotline 12345 was designed to handle social affairs such as policy consultation, complaints and help, suggestions and feedback, and to smoothen the channels for people to express their demands^6^. It is a bottom-up, audience-centered grassroots approach that aims to factor the public’s opinion into the campaign development and policy-making process. After the implementation of comprehensive smoke-free legislation, the hotline 12345 platform also serves as an important way for the public to participate in activities such as monitoring illegal smoking behaviors.

### Study aims

**Figure 1:**
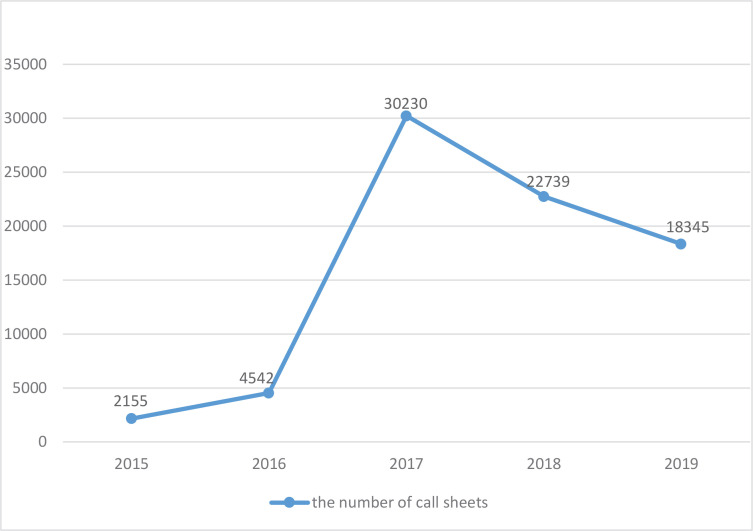
Descriptive statistical analysis of the call sheet number on smoking issues. The statistic results showed fluctuations between 2015 to 2019 and a quantity peak in 2017, rising in line with the time when the Amendment was implemented

We propose to make recommendations on smoke-free legislation by collecting and analyzing a large amount of feedback from the public supervision activities. Here, we introduced a feasible method to decode the information of the hotline 12345 call sheets. As they recorded the residents’ viewpoints on tobacco control issues, and notably, their number fluctuated significantly after the announcement of the new policy (Figure 1), our study should be helpful in answering the following questions:

1. How did residents initially respond to the implementation of the Amendment?
2. How well does the implementation of existing policies fit in the citizens’ demands for smoking bans?
3. How can information extracted from calls uncover the issues of greatest concern to citizens and the locations with the most feedback, thereby optimizing the allocation of smoking control monitoring resources, reducing the costs of smoking control enforcement, and contributing to future tobacco control decision-making?

## METHODS

### Overview

As the hotline 12345 not only received smoking cessation counseling but any advice related to tobacco control since it was launched five years ahead of the Amendment, the platform was enriched with numerous data, including a large amount of text content. To make better use of the information embedded in call sheets, we will apply text-mining technology, a frequently-used method for text analysis on social media, news reports, and the literature^7-11^.

### Design

Of the various data types, text is the most unstructured form, and without processing, text data cannot be analyzed by a computer. Text mining technology has been gradually applied to literature analysis and public opinion analysis^12^. Therefore, when dealing with text data, relevant programming languages have been used for text analysis and text mining. As for the research design, we mainly refer to previous research that had collected tweets from Twitter to study electronic cigarette regulations^13^. They performed a descriptive text-mining, which has been of great value for our current research on tobacco control legislation from public demands.

Text mining mainly uses a natural language processor to clean up and create word dictionaries. In the Chinese language environment, the Chinese word segmentation tool is essential^14^. Meanwhile, the application of topic modeling has made it easier to extract the main ideas of the text and facilitate analysis^15^.

### Data collection

The service management application system of hotline 12345 was responsible for providing overall systemic support for the daily workflow. Voice conversations were recorded by the hotline’s management system and converted into text by telephone operators to collect information on residents’ appeals^16^. All text documents were written to hotline transcripts, which can be called call sheets.

**Figure 2:**
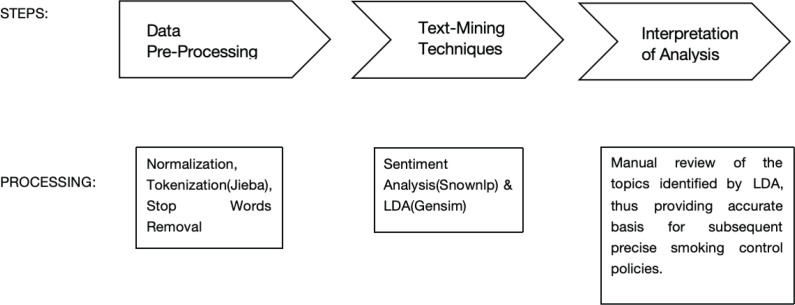
Schematic diagram of research

We collected 78011 call sheets related to tobacco control from the past five years (1 January 2015 to 31 May 2019), all of which are to be analyzed using text mining (Figure 2) and descriptive statistics. Due to the pandemic, data post-2019 have not been made public by the government. Currently, we are requesting the government to release these data.

### Every call sheet contained five items:

1. Identification: the time and sequence number were generated for every call sheet, such as ‘20170301005078’. This number could be used to identify and record the specific time and date of the call^17^.
2. Location: The location where the smoking behavior occurred.
3. Type: The contents of the calls were divided into five categories: complaint, help-seeking, consultation, suggestion, and others.
4. Descriptive information: This item contained a context-specific description of the calls, such as the details of the reported problems, the caller’s appeals, and the replies from the call center.
5. Feedback: It recorded the subsequent processing results from relevant government departments.

### Procedure

*Stage 1: Classification of data from call sheets*

Initially classified by research problems, the data in our study are to be re-analyzed according to separate items. This step is particularly crucial due to the unique expression and record habits of time, place, and specific events in the Chinese language. To address these nuances, researchers will employ methods such as Chinese word segmentation and word embedding to effectively clean and extract fields, specifically focusing on Chinese words. Additionally, our analysis will utilize a Sentiment Lexicon specifically designed for the Chinese language. This lexicon plays a pivotal role in assigning sentiment scores to words based on their connotative meaning, thereby aiding in the identification of positive, negative, and neutral sentiments expressed in the text.

*Stage 2: Content analysis for different call sheets*

The amendment not only expanded the number of non-smoking places but also made it clear that non-smoking signs should be put up in public areas, including restaurants and workplaces, and ashtrays should not be provided in all indoor places. We wish to know how the residents reacted to the new regulations (the Amendment) and how were the responses and feedback from the relevant government departments. This required clustering statistics of call sheets with different contents by using topic model, such as ‘Latent Dirichlet Allocation’ and ‘k-means clustering algorithm’. In addition to our two-staged descriptive statistical analysis, we integrate Latent Dirichlet Allocation (LDA) within a Bayesian framework for advanced topic modeling. LDA, through its probabilistic Bayesian method, accurately infers the distribution of topics, allowing us to analyze shifts in public perception and discussion, particularly around key policy events. LDA will be employed to uncover latent topics within the dataset. We will explore a range of topic numbers and chose the optimal model based on coherence scores, which measure the semantic similarity between the high-scoring words in each topic. The reliability of our topic model will be validated through a two-step process. Initially, a subset of data will be manually categorized to create a benchmark. The model’s output will then be compared against this benchmark to assess accuracy and adjust parameters accordingly. Based on initial findings, the model will undergo several iterations of refinement, adjusting parameters such as the number of topics and token filters to better align with the research objectives and improve interpretability of the results.

*Stage 3: Advice on policies*

We will conduct a comprehensive analysis of the issues most frequently complained about by citizens in the work orders, as well as the locations of these complaints, and compare them with the findings of tobacco control studies in other countries. Additionally, we will utilize LDA to uncover latent topics within our textual data, enhancing our understanding of public sentiment and the impact of policies. This combination of statistical and topic modeling techniques not only enriches our analysis but also allows us to effectively compare situations before and after significant policy milestones, leading to well-informed recommendations. By integrating real-time smoking control monitoring data from local disease control departments, we will verify that our research conclusions are both universal and effective. Our approach, aligning detailed statistical insights with nuanced thematic understanding from LDA, is comprehensive and adept at pinpointing policy deficiencies and proposing targeted improvements. The identified topics will be interpreted by analyzing each topic’s most representative words and documents. Themes will then be named and categorized into broader categories reflecting public concerns, sentiment, and suggestions regarding tobacco control policies. This will enable us to make targeted smoking control suggestions to optimize the allocation of human resources during the smoking control process, reduce enforcement costs, and provide guidance for the government to clarify directions for improving smoking control measures.

## DISCUSSION

In order to answer the above research questions, in addition to using data as evidence, we still need to add more investigation and research materials. To expand on interviews and qualitative content analysis, we need to compare the results of previous surveys on residents’ reactions and the implementation and optimization of existing policies so as to achieve our research purpose. Furthermore, in collaboration with the local CDC, we plan to incorporate COVID-19 data into our analysis. These additional data will help us understand the intersection between tobacco control measures and pandemic responses, potentially highlighting new areas for policy enhancement and resource allocation.

The main purpose of our research is to provide constructive suggestions for existing policies by examining information from call sheets. Policy makers could use hotline information to learn the public’s concerns and viewpoints on the Amendment.

In our research, the original voice data that came from hotline 12345 calls, were converted into text data, serving as objective records of residents’ real feedback on Shanghai’s newly launched smoke-free legislation. More attention was paid to exploring the utilization of hotlines in health education and thus to promoting people’s health awareness and behaviors, as shown in previous studies on hotlines. And some researchers may even ask specific questions via hotlines to make it much more like a telephone survey to get the information they need^18-20^. Therefore, this study introduces hotline analysis based on text mining, which uncovers the issues most concerning to citizens and the locations receiving the most feedback. This enables the optimization of resource allocation for smoking control monitoring, reduces the costs of smoking control enforcement, and contributes to future decisions regarding smoking control policies.

When considering the issue of changing trends, we may need to conduct a retrospective analysis. As early as 2010, before the Regulation was completed and revised, several surveys and studies on residents’ reactions to tobacco control policies were conducted in Shanghai, suggesting the strengthened public promotion and education efforts to raise awareness and understanding of the Regulation among the residents^21-24^. Several studies indicated that audience-centered education programs and health awareness campaigns influence residents’ participation in hotline supervision. In a study of public health campaigns and their impact on health behavior in the United States, their research indicated that viewing some help-seeking-related messaging may increase saliency or awareness of the availability of hotlines for help^20^. An investigation in 2009 showed that the smoking groups in China were mainly working populations characterized by group clustering^22^. So, diverse protocols targeting different subpopulations also need to be developed to provide better services to the public. However, this population has changed over the decades, which requires us to update the latest information on the characteristics of smokers. Related to this, the change in the correlation between smoking and places is also a focus of the study. Evidence has shown that after the carry-out of the Amendment, compliance was the weakest in farmer’s markets and bars, and the smoking behaviors in male toilets did not change significantly, which implied that the enforcement conditions vary with the particularity of places^23^. When enforcing the law, accurate smoke control policies should be implemented according to the particularity of different sites^24^. The factors related to these changes after the amendment was enacted in 2017, remain to be answered.

Moreover, our study aligns with global trends in tobacco use and control. For instance, it mirrors situations in countries like Pakistan^25^, where smoke-free policies in government offices in major cities are inadequately enforced. Our analysis identifies workplaces and dining establishments as key concerns, with significant percentages of work orders originating from these locations. This highlights the necessity for targeted tobacco control surveillance in office buildings, restaurants, and residential areas.

To further validate our research findings, we are planning collaborations with relevant government public health monitoring departments for future studies. This will involve targeted inspections at locations frequently mentioned in our research, followed by a comparison of these inspection results with subsequent citizen call data. Such steps are pivotal to ensuring that our findings are not just theoretically robust but also practically applicable in shaping policy-making. In summary, while national smoke-free legislation has not yet been launched in China, our study, leveraging text-mining technology in analyzing hotline data, aims to enhance existing policies and contribute valuable insights to tobacco control legislation

### Future research and considerations

In future studies, we plan to use more extensive datasets, enabling us to further refine our insights and recommendations. This continual expansion of data sources will allow us to constantly update and improve our strategies for effective tobacco control and public health initiatives. To enhance the robustness and applicability of our methodology, future research could include a retrospective analysis of changing trends and public attitudes. Examining past data and trends can help identify factors that may have influenced public behaviors and attitudes at different points in time. By integrating data from the Centers for Disease Control and Prevention (CDC) on tobacco control monitoring, along with conducting real-time situational surveys, we will be able to understand better and address the dynamic changes in tobacco use. This approach can provide critical insights for developing more effective public health strategies. Additionally, investigating shifts in smoking behavior, especially in correlation with different settings, can offer a deeper understanding of enforcement challenges and the need for context-specific strategies.

Exploring the changing demographics of smoking groups and the evolving dynamics of public spaces where smoking occurs, will further enrich the effectiveness of tobacco control measures. By incorporating demographic information from tobacco control tickets, we can regularly update the characteristics of smokers and adapt our nuanced enforcement strategies to suit various settings. This approach ensures that policies maintain their continued relevance and impact over time.

### Limitations

While our methodology offers substantial insights, it is essential to acknowledge potential limitations associated with data bias, accuracy, and completeness. Firstly, due to the increased sensitivity of data during the pandemic period, our research team is actively engaging with relevant government departments to gain access to these sensitive datasets. The absence of these data could limit the comprehensiveness of our analysis in capturing recent public sentiments and responses, potentially affecting the study’s relevance to current public opinions and the effectiveness of existing policies. Secondly, while our substantial dataset and rigorous methodology allow us to suggest broader applicability, our study is inherently limited to a specific geographical and demographic area. The patterns and behaviors regarding public sentiment and smoking regulations that we identified may not fully represent global trends. This is particularly relevant when considering extrapolation to countries with differing health policies and smoking cultures from those studied. Lastly, while our study offers valuable insights into tobacco control policy in Shanghai, its generalizability to other countries or regions is limited due to cultural, social, and legislative differences. However, the methodology and findings may still serve as a valuable reference for countries with high smoking rates or developed nations, providing a framework for adapting similar strategies to their unique contexts.

Our methodology harnesses text mining and topic modeling to extract valuable insights from call transcripts, shedding light on public perceptions and attitudes towards tobacco control policies. While the data source may present certain limitations, the robustness of our approach contributes significantly to enhancing policy-making and public health awareness. Future research efforts are needed to explore these aspects further and to update the dataset with more recent information post-pandemic to enhance the study’s comprehensiveness and applicability.

## CONCLUSIONS

Our research provides key insights for refining tobacco control policies and introduces a methodology that can be applied to a range of public health challenges, and we have proposed specific measures in the areas of enhanced supervision, law enforcement, and public education. By leveraging public feedback and utilizing text-mining techniques, policymakers can formulate strategies that are more informed and effective. This approach is particularly vital as China progresses towards comprehensive national health improvements.

## Data Availability

The data supporting this research are available from the authors on reasonable request.
